# Long‐Term Outcome of Guided Crown Lengthening and Lip Repositioning in the Treatment of Excessive Gingival Display

**DOI:** 10.1155/crid/7330406

**Published:** 2026-04-16

**Authors:** Hytham N. Fageeh

**Affiliations:** ^1^ Department of Preventive Dental Sciences, College of Dentistry, Jazan University, Jazan, Jazan Province, Saudi Arabia, jazanu.edu.sa

**Keywords:** altered passive eruption, case report, crown lengthening, digital surgical guide, excessive gingival display, lip repositioning

## Abstract

**Background:**

Managing excessive gingival display (EGD) is challenging and requires precise diagnosis and etiology‐driven treatment for long‐term stability. This report describes a digitally guided, staged approach combining esthetic crown lengthening (ECL) and lip repositioning (LR) for multifactorial EGD.

**Case Presentation:**

A 28‐year‐old female presented with 7 mm of gingival display on dynamic smile, upper lip length of 19 mm, and lip mobility of 6 mm. The etiologic diagnosis was vertical maxillary excess (VME) Class II, hyperactive upper lip (HUL), and altered passive eruption (APE) (Coslet Type 1 B). Digital planning was performed using 3Shape intraoral scanning integrated with digital smile design (DSD) to establish target gingival zeniths and smile line. A dual‐purpose three‐dimensional (3D)‐printed surgical guide (Form 3, Formlabs) was designed to (1) control the apical extent of crown lengthening and (2) visualize the anticipated dynamic lip line, targeting a residual gingival display of 2–3 mm.

**Management and Outcome:**

Phase 1 involved ECL with gingivectomy and osseous recontouring to reestablish biologic width and create ideal crown proportions. After 6 weeks of healing, LR was performed following Bhola’s technique with a partial‐thickness mucosal excision, selective myectomy, and deep horizontal mattress sutures for stabilization. Gingival display reduced from 7 mm at baseline to 0 mm at 3 months, and measured ~2–3 mm after 5 years of follow‐up, maintaining a pleasing smile arc and high patient satisfaction.

**Conclusion:**

A digitally guided staged approach integrating ECL and LR effectively managed combined EGD etiology. The 3D‐printed guide enhanced surgical precision and predictability, with sustained esthetic improvement at 5 years and minor recurrence (2–3 mm).

## 1. Introduction

A primary goal of periodontal plastic surgery is to achieve optimal esthetic outcomes for the patient’s smile. However, certain patients presenting with both gingival and skeletal deformities may necessitate more comprehensive esthetic rehabilitation. For patients with complex needs, a multidisciplinary approach is advantageous in optimizing the balance and integration of all three elements of the smile, teeth, lip framework, and gingival scaffold [1].

The ideal gingival display during smiling is generally considered to be ~1–3 mm [2]. Excessive gingival display (EGD), also referred to as a “gummy smile,” is an important component of smile analysis and a common cause of esthetic dissatisfaction. It is defined as excessive exposure of gingival tissue during smiling [3]. Reported etiologies involve both hard‐ and soft‐tissue factors, including altered passive eruption (APE), anterior dentoalveolar extrusion, vertical maxillary excess (VME), and a short or hyperactive upper lip (HUL) [2, 4]. Multiple coexisting etiologies are common; therefore, accurate identification of the underlying factors is essential for treatment planning and prognosis [1].

APE is characterized by incomplete apical migration of the gingival margin following tooth eruption, resulting in short clinical crowns and excessive gingival coverage despite normal anatomic crown dimensions [5]. Coslet et al. [6] proposed a classification of APE based on the relationship of the gingival margin, keratinized tissue, and alveolar crest to the CEJ, which guides treatment planning. The type of treatment proposed for any clinical situation of APE depends upon the given classification [7]. When APE occurs in combination with a HUL or mild VME, a staged surgical approach is often indicated, which includes minimally invasive periodontal and soft‐tissue procedures that effectively enhance esthetics [8, 9].

The first phase generally involves esthetic crown lengthening (ECL), which aims to reestablish the proper biologic width (~2–3 mm apical to the CEJ) and create stable gingival margins that accurately define the clinical crown proportions [9]. Performing ECL before lip repositioning (LR) is essential, as it allows soft‐tissue maturation, ensures periodontal health, and prevents unpredictable margin rebound that could compromise the symmetry of the subsequent lip surgery [3]. The second phase, LR, is then planned relative to the newly established gingival line, targeting optimal harmony between dental and facial esthetics.

Advances in digital smile design (DSD) and three‐dimensional (3D) printing have significantly improved the precision of such interdisciplinary treatment planning [10, 11]. Digital integration facilitates visualization of both gingival and lip dynamics, enabling the fabrication of customized dual‐purpose surgical guides that define the exact apical limit of crown lengthening while simultaneously predicting the anticipated dynamic lip line during a smile [10, 11]. This workflow enhances surgical accuracy, predictability, and communication between the clinician and patient.

This case report describes a digitally guided (scan → DSD → 3D‐printed guide), staged management approach involving ECL followed by LR, with a 5‐year clinical follow‐up, highlighting the use of a dual‐purpose surgical guide that precisely defines the extent of gingival recontouring while simultaneously visualizing the anticipated dynamic upper‐lip line for enhanced esthetic predictability.

## 2. Case Report

A 28‐year‐old female presented to the College of Dentistry, Jazan University, with the chief complaint of EGD during smiling and laughter. Her medical history was noncontributory, with no relevant medications or allergies. The patient had completed orthodontic treatment 2 years prior to presentation.

### 2.1. Clinical Examination

**Figure 1:**
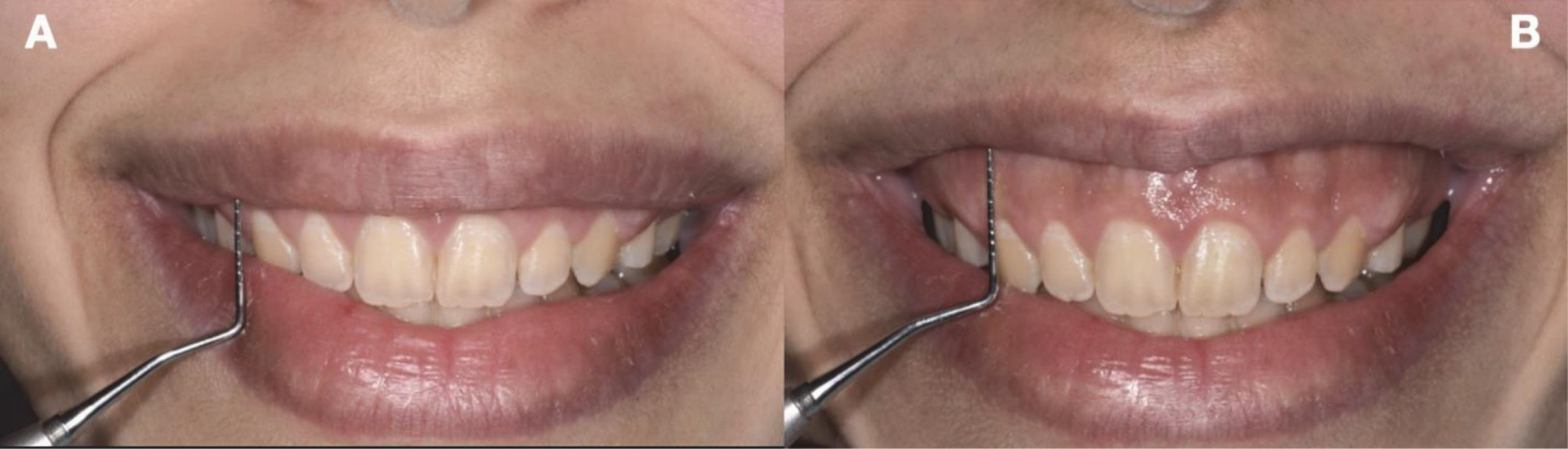
Baseline extra oral views. (A) Smile in posed/static position with 1 mm of gingival display. (B) Smile in dynamic position with 7 mm of gingival display.

Extraoral assessment revealed an upper lip length of 19 mm from the base of the nose to the stomion superius at rest. On a posed/static smile, 1 mm of gingiva was visible (Figure 1A), whereas a spontaneous/dynamic smile revealed 7 mm of gingival display, indicating 6 mm of upper‐lip mobility (Figure 1B). Intraorally, the patient exhibited a thick gingival biotype and a keratinized tissue width of 8–11 mm across the maxillary anterior sextant, with mild bilateral bony exostosis. Disproportionate crown height‐to‐width ratios were noted.

Bone sounding at tooth #13 under local anesthesia revealed the alveolar crest 3 mm apical to the gingival margin, confirming APE (Coslet Type 1B). Periapical radiographs were taken to evaluate the CEJ‐crest relationship and root morphology. The case was further classified as VME Class II with a HUL pattern. The diagnosis integrated both skeletal and soft‐tissue etiologies of a “gummy smile.”

### 2.2. Treatment Planning

After discussing treatment options, informed consent was obtained for a two‐phase approach: (1) ECL followed by (2) LR after 6 weeks of soft‐tissue healing.

**Figure 2:**
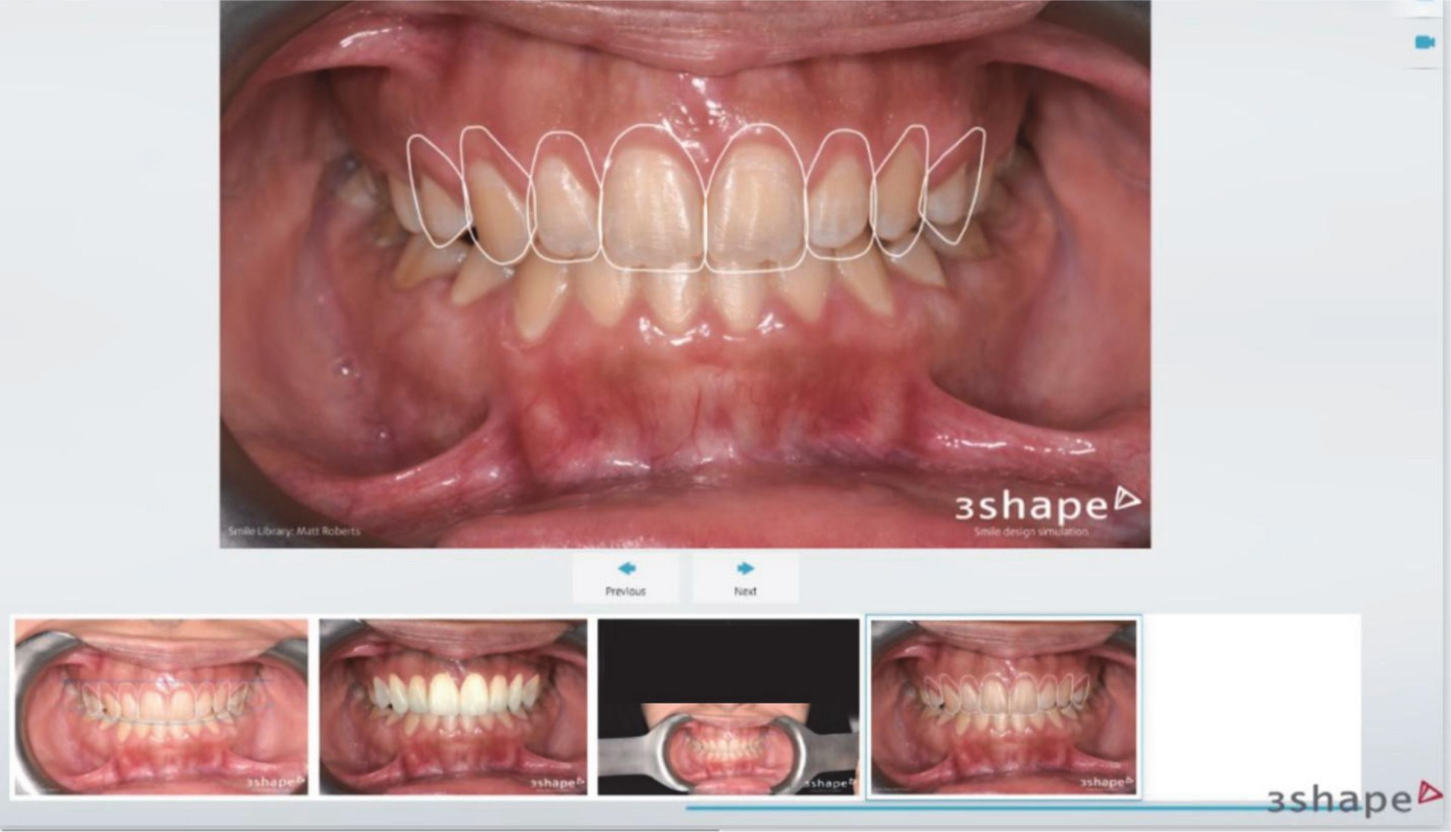
Digital smile design to determine adequate crown height/width ratio.

Digital preoperative photographs were imported into 3Shape DSD software to establish the ideal gingival zeniths and marginal levels (Figure 2).

**Figure 3:**
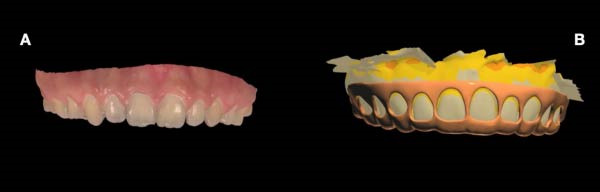
(A) Baseline intraoral scan of the maxillary arch. (B) Dual‐purpose surgical guide on virtual wax‐up of teeth based on digital smile design.

Guide fabrication was carried out following intraoral scanning of both the maxillary and mandibular arches using a Trios 3 scanner (3Shape, Denmark) (Figure 3A). A dual‐purpose 3D‐printed surgical guide was subsequently designed based on a virtual wax‐up generated from the DSD workflow. The guide incorporated (1) an apical reference to determine the precise extent of ECL and (2) a horizontal terminal border corresponding to the anticipated dynamic smile upper‐lip line, allowing for a residual gingival display of ~2–3 mm posttreatment (Figure 3B).

**Figure 4:**
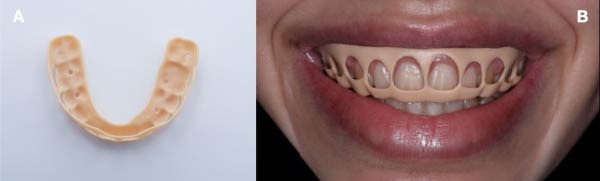
(A) 3D‐printed guide for esthetic crown lengthening. (B) Intraoral seating of the surgical guide.

The guide was printed and postprocessed per manufacturer instructions using a 3D printer (Form 3, Formlabs, USA) (Figure 4A) and verified in the patient for passive seating (Figure 4B).

### 2.3. Therapeutic Intervention

**Figure 5:**
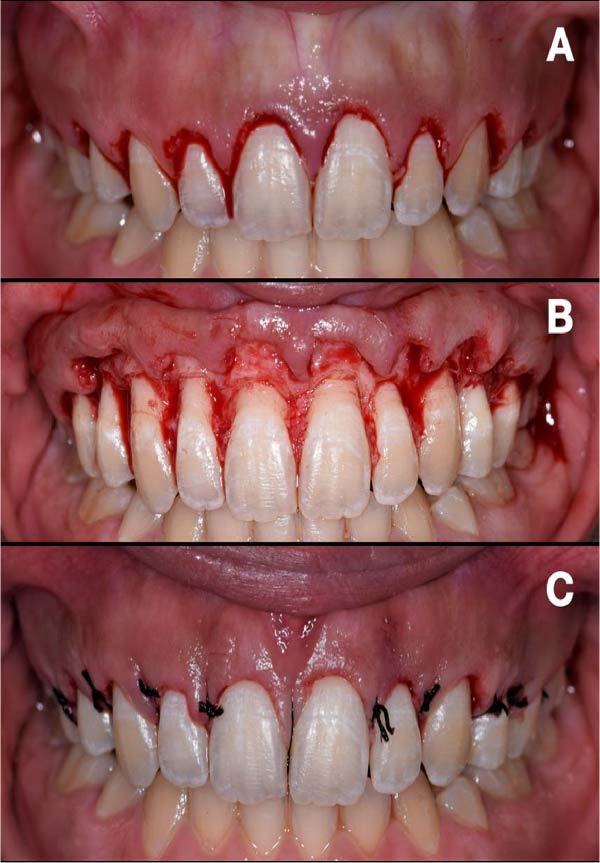
(A) Gingivectomy by external bevel incision following crown outline on the surgical guide. (B) Osseous resection of alveolar crest to reestablish the biologic width, interdental papillae deepithelialized using curved micro scissors. (C) Flap repositioned and sutured with 4.0 (silk) sutures.

Phase 1: ECL: After intraoral disinfection with 0.2% chlorhexidine, local anesthesia was administered via infiltration of the maxillary arch using 2% mepivacaine with 1:100,000 epinephrine. With the surgical guide properly seated, gingivectomy was performed using an external bevel incision made with a No. 15C blade, following the DSD‐defined soft‐tissue margins and using the guide as a visual reference (Figure 5A). A full‐thickness mucoperiosteal flap was then reflected from the upper right to upper left maxillary molar to facilitate osseous resection and osteoplasty, reestablishing the biologic width ~3 mm apical to the CEJ, as verified with a periodontal probe. Osteoplasty was performed on the buccal aspect using a multifluted bur, whereas osteotomy was accomplished with an end‐cutting bur to minimize the risk of tooth or root surface damage.

Root planing was performed to reduce tissue rebound and facilitate soft‐tissue adaptation. The interdental papillae were deepithelialized using curved micro scissors (Figure 5B). Thereafter, the flap was repositioned and sutured with 4–0 silk using single interrupted sutures engaging the palatal aspect of each papilla (Figure 5C). No periodontal dressing was applied.

Postoperative instructions included applying ice packs bilaterally for 2 days (10‐min sessions twice daily) and cleansing the sutures with gauze soaked in 0.2% chlorhexidine after toothbrushing for 1 week. The patient was advised to maintain a soft and cold diet for 2 days and was counseled on modified oral hygiene measures. Ibuprofen (600 mg every 8 h as needed) was prescribed for analgesia.

**Figure 6:**
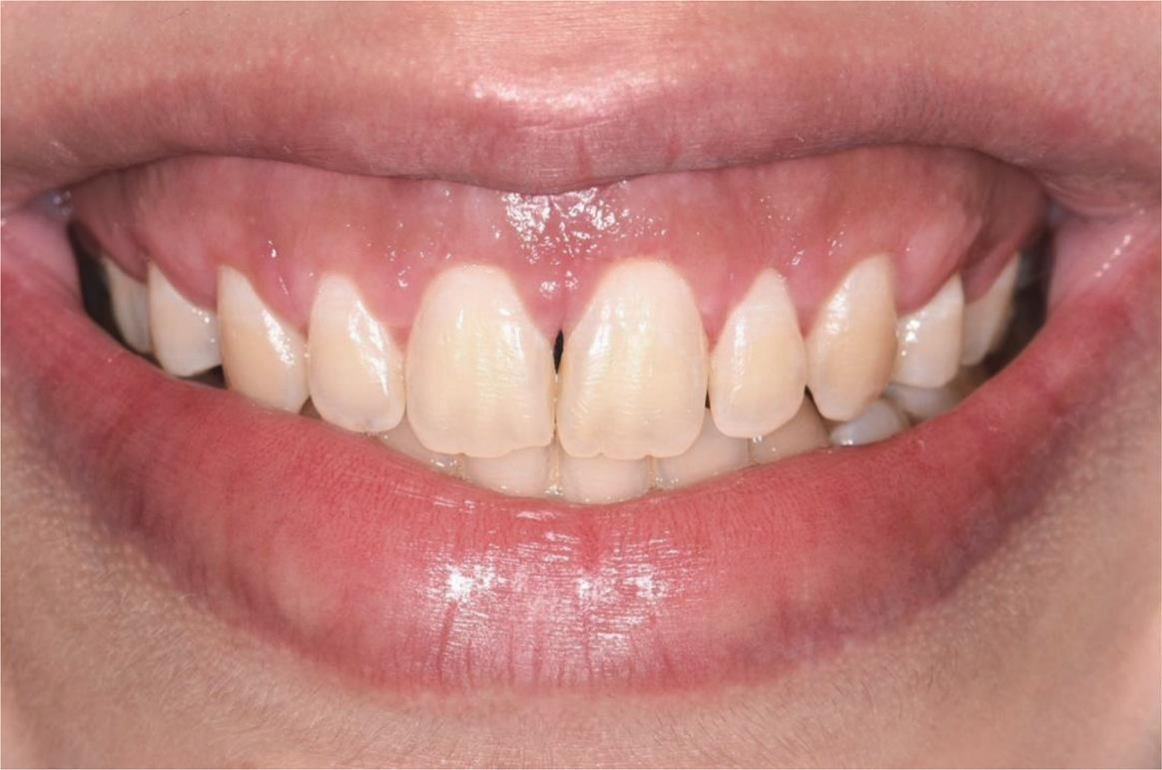
Frontal view of healing at 6 weeks following esthetic crown lengthening.

After 10 days, all sutures were removed. The patient reported minimal discomfort, with no evidence of swelling or edema. Satisfactory gingival healing and an observable gain in clinical crown height were noted. A healing period of 6 weeks was allowed to permit soft‐tissue maturation and margin stabilization before proceeding with LR surgery. At the follow‐up visit, the gingival tissues appeared stable, and probing depths remained within normal limits (Figure 6).

**Figure 7:**
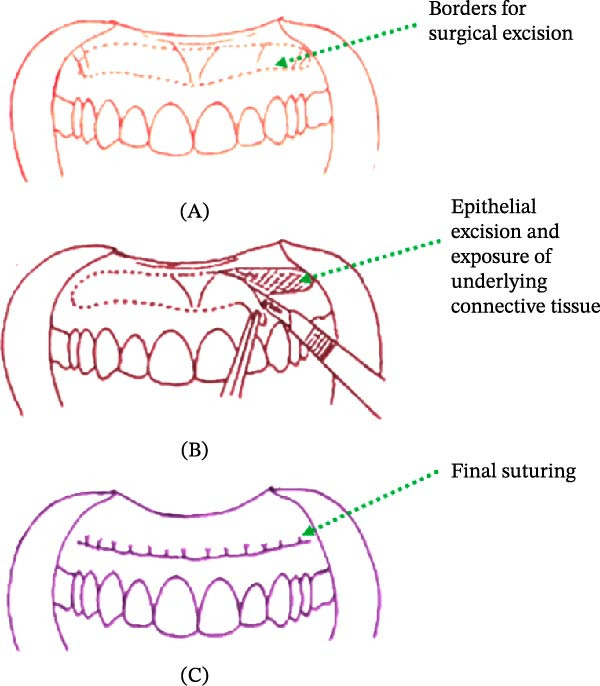
Schematic representation of lip repositioning surgery. (A) Marking borders for surgical excision. (B) Epithelial excision and exposure of underlying connective tissue. (C) Final suturing (made on Microsoft PowerPoint).

**Figure 8:**
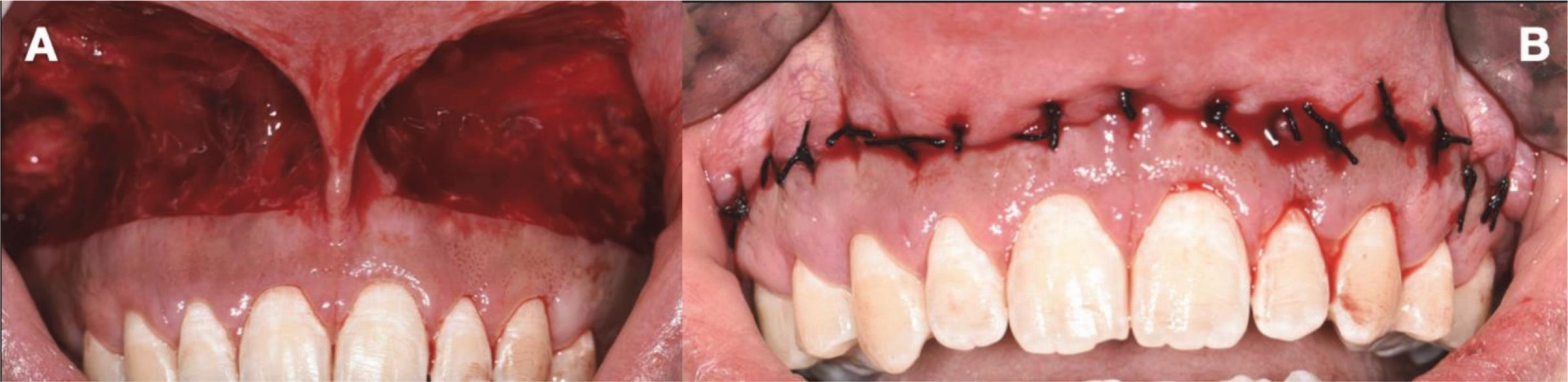
(A) Intraoral view following excision of the mucosal strip and partial‐thickness flap. (B) Simple interrupted sutures with 4.0 silk approximating the upper and lower border for primary intention healing.

Phase 2: LR: The LR procedure was performed according to the technique described by Al Jasser et al. [12] (Figure 7). The preoperative anesthetic protocol included buccal vestibular infiltration and infraorbital nerve block using 2% mepivacaine with 1:100,000 epinephrine. The excision outline was marked with a No. 15C scalpel blade, extending between the maxillary molars while preserving an adequate band of attached gingiva along the mucogingival line (Figure 7B). The superior margin of the excision was delineated parallel to the inferior margin at a distance corresponding to twice the measured upper‐lip mobility between the static and dynamic smile positions (Figure 8A). Specifically, the vertical distance between the upper and lower borders of the rectangular outline measured 12 mm.

A partial‐thickness mucosal strip was excised along this outline to expose the underlying connective tissue. A selective myectomy of the elevator fibers of the levator labii superioris was performed to reduce excessive superior lip retraction. Muscle separation was completed with the help of blunt dissection, and the muscle fibers were pushed upward, leaving underlying periosteum intact. The displaced muscle fibers were trimmed to eliminate any remaining pull. Hemostasis was achieved primarily through direct pressure with sterile gauze. Minor bleeding points were controlled using low‐power electrocautery in a spot‐coagulation mode, ensuring minimal thermal damage to surrounding tissues. The surgical field was thoroughly irrigated with sterile saline before flap repositioning and suturing.

Deep horizontal mattress sutures with 5–0 chromic gut were placed at predetermined intervals to stabilize the lip in a slightly inferior position and minimize relapse. These were supplemented by 4–0 silk simple interrupted sutures placed along the incision line (Figure 8B). The labial frenum was preserved throughout the procedure to maintain midline alignment and was repositioned only during suture placement.

#### 2.3.1. Postoperative Instructions

Postoperative medications included a corticosteroid regimen of prednisone (5 mg, two tablets three times daily for the first 3 days, followed by one tablet three times daily for days four and five) and diclofenac potassium (50 mg every 8 h as needed). Postsurgical instructions emphasized the application of ice packs, avoidance of exaggerated facial expressions or lip elevation for 14 days, adherence to a soft diet, and cleansing with gauze soaked in 0.2% chlorhexidine for 1 week following toothbrushing.

**Figure 9:**
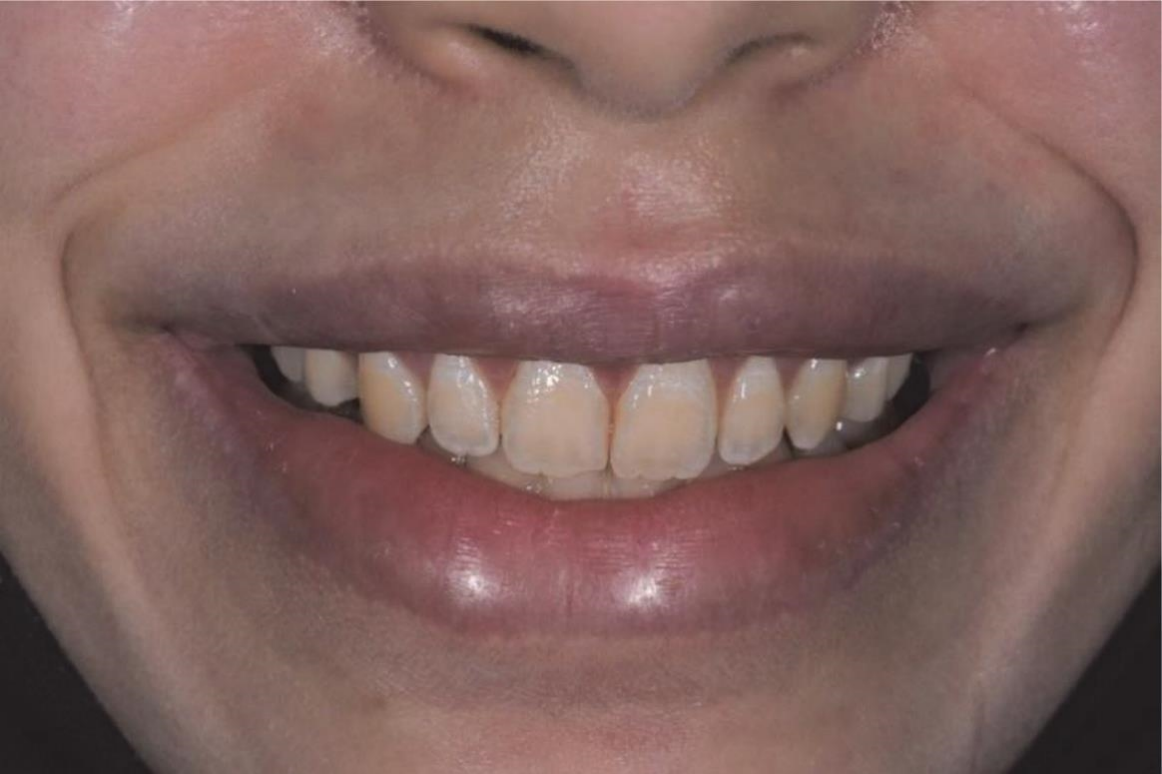
90 days’ postoperative period following lip repositioning procedure.

At the 1‐week follow‐up, mild discomfort and tightness were noted, with limited upper‐lip motion. Sutures were removed after 14 days. Gingival display was completely reduced in the early postoperative period, and at 1‐ and 3‐month follow‐ups, no relapse was evident with 0 mm gingival display at 3 months (Figure 9).

### 2.4. Outcome and Follow‐Up

**Figure 10:**
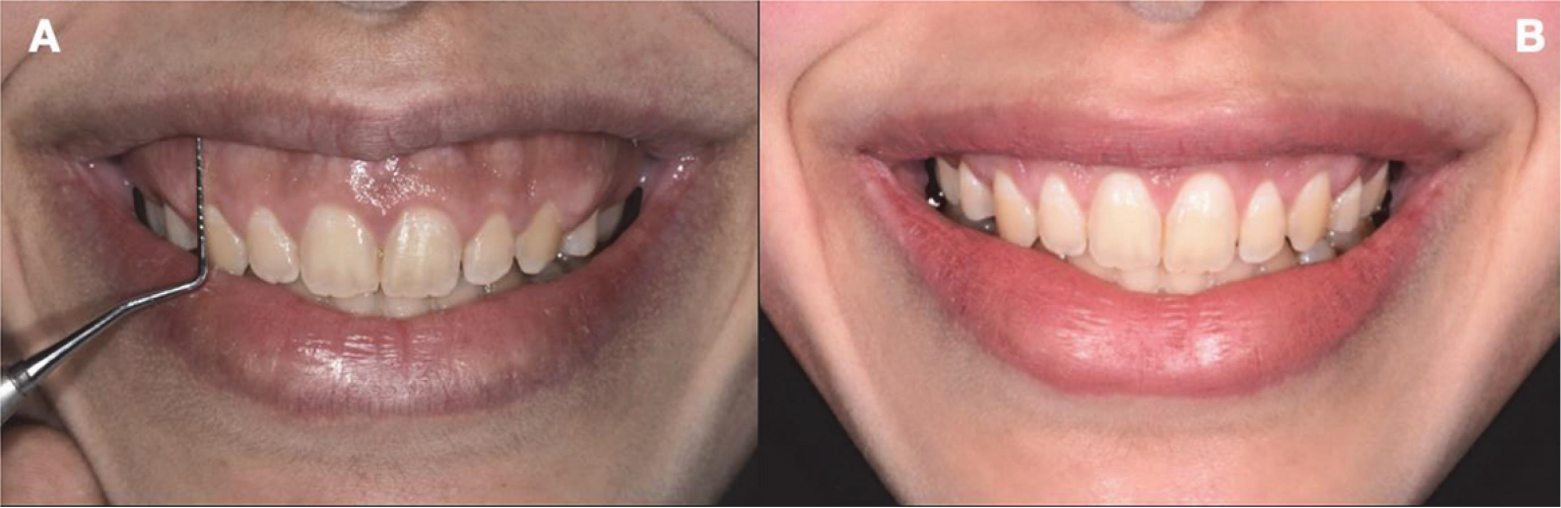
Frontal view. (A) Initial appearance in dynamic smile. (B) Appearance in dynamic smile after 5 years of follow‐up.

At the 5‐year follow‐up, the crown height‐to‐width ratio achieved by ECL remained stable. However, partial relapses of upper‐lip position were observed during dynamic smiling (Figure 10B). At the 5‐year follow‐up, gingival display measured 2–3 mm, indicating a 2–3 mm partial relapse compared to the 3‐month outcome. Despite this minor recurrence, the patient expressed high satisfaction with the esthetic and functional outcomes. Written informed consent was obtained from the patient for publication of the clinical details and accompanying images. Table 1 demonstrates clinical stages and outcomes measured during the complete treatment procedures.

**Table 1:** Chronological timeline of diagnosis, surgical phases, and follow‐up.

Timepoint	Clinical stage	Key findings/procedures	Outcome
Initial visit	Diagnosis and baseline assessment	28‐year‐old female with excessive gingival display; 1 mm (posed smile), 7 mm (dynamic smile); 6 mm lip mobility; Coslet Type 1B altered passive eruption; VME Class II with hyperactive upper lip	Two‐phase treatment planned
Presurgical planning	Digital smile design and guide fabrication	DSD performed using 3Shape software; intraoral scan with Trios 3; dual‐purpose 3D‐printed surgical guide fabricated	Ideal gingival zeniths and surgical limits established
Phase 1–day 0	Esthetic crown lengthening (ECL)	External bevel gingivectomy; full‐thickness flap; osteoplasty and osteotomy; biologic width reestablished (3 mm apical to CEJ); sutured with 4–0 silk	Uneventful procedure
10 days post‐ECL	Suture removal	Clinical healing satisfactory; increased crown height observed	Soft‐tissue healing progressing normally
6 weeks post‐ECL	Pre‐lip repositioning assessment	Gingival tissues stable; probing depths within normal limits	Cleared for Phase 2 surgery
Phase 2–week 6	Lip repositioning (LR) with selective myectomy	Partial‐thickness mucosal excision (12 mm vertical height); selective myectomy of elevator fibers; deep horizontal mattress (5–0 chromic gut) + simple interrupted sutures (4–0 silk)	Immediate reduction in lip mobility
1 week post‐LR	Early healing review	Mild discomfort; tightness; limited lip motion	Normal postoperative healing
2 weeks post‐LR	Suture removal	Stable lip position; improved smile esthetics	Gingival display reduced to 0 mm
1 month post‐LR	Short‐term follow‐up	No signs of relapse	Stable outcome
3 months post‐LR	Short‐term stability assessment	Gingival display maintained at 0 mm	No relapse observed
5 years post‐LR	Long‐term follow‐up	Gingival display measured 2–3 mm (2–3 mm gingival relapse from the 3‐month result [0 mm]); stable crown proportions	High patient satisfaction; gingival display reduced to 2–3 mm; overall 4–5 mm reduction from baseline

## 3. Discussion

EGD, commonly referred to as a “gummy smile,” is a multifactorial esthetic concern that may arise from a combination of skeletal, dental, and soft‐tissue factors. Among these, VME, APE, and HUL movement are frequently reported etiologies [13]. A comprehensive diagnostic assessment is thus essential for accurate classification and individualized treatment planning. LR was originally described by Kostianovsky and Rubinstein [14] in 1976 as a surgical approach for reducing EGD. In the present case, the patient exhibited features of APE (Coslet Type 1 B), VME (Class II), and HUL mobility, necessitating a multidisciplinary approach involving periodontal and mucogingival corrective procedures.

DSD can enhance esthetic treatment planning by integrating facial and dental parameters into a reproducible workflow [15]. The use of 3Shape’s DSD software in this case enabled precise identification of gingival zeniths and target margin levels, providing both the clinician and patient a visual simulation of the expected outcome. Furthermore, the fabrication of a dual‐purpose 3D‐printed surgical guide served as an innovation in controlling the vertical extent of gingivectomy and correlating the lip line with the anticipated smile arc. Such digital integration has been shown to increase accuracy in gingival margin repositioning and improve postoperative esthetic predictability [15–17].

The first therapeutic phase, ECL, aimed to correct the APE and establish ideal crown proportions. According to the biologic width/supracrestal tissue dimension concept, ~2–3 mm between the alveolar crest and gingival margin is required for periodontal health [2]. In this case, osteoplasty and osteotomy were performed to reestablish this dimension, preventing tissue rebound. A 6‐week healing interval was selected to allow initial soft‐tissue healing and clinical reassessment before LR; however, gingival margin remodeling after crown lengthening may continue beyond this period, and long‐term margin stability should not be inferred from early healing alone [18–20].

The second phase of LR targeted the hypermobile muscular component of the upper lip. Originally described by Kostianovsky and Rubinstein [14], the procedure has since undergone several modifications aimed at reducing relapse, optimizing symmetry, and minimizing postoperative discomfort [21, 22]. In this case, the modification described by Al Jasser et al.. [12] was used, which limits the superior extent of mucosal excision to twice the measured lip mobility and preserves the frenum for midline stability. The addition of a selective myectomy of the levator labii superioris was performed to reduce the elevator muscle pull, a step that has been associated with improved long‐term stability in some reports [3, 21, 22].

Postoperative outcomes demonstrated a marked reduction in gingival display from 7 mm at baseline to 0 mm at 3 months post‐LR, with 2–3 mm gingival display at the 5‐year follow‐up, achieving a harmonious smile line. Early follow‐ups revealed minimal discomfort, confirming the minimally invasive nature of the approach. The patient expressed high satisfaction both esthetically and functionally. These outcomes align with prior literature showing positive patient‐reported satisfaction and improved smile esthetics following LR [15, 16, 19, 20].

Long‐term stability remains a challenge in the management of EGD. Several studies have documented partial relapses in gingival display postoperatively, attributed primarily to muscle reattachment or incomplete fibrosis at the resection site [3, 19–22]. In this report, a mild degree of relapse was noted at the 5‐year follow‐up, although the crown lengthening results remained stable. This finding reinforces that LR, while effective, may not be permanent and requires careful patient selection and realistic expectations. Adjunctive approaches, including modified surgical techniques and myotomy, have been explored to enhance longevity but require further evidence [1, 23]. In a systematic review by Ardakani et al. [1], it was concluded that minimal relapse occurs in the selective myotomy surgeries. In randomized data, outcomes have been compared with and without myotomy, with relapse reported more frequently in non‐myotomy groups [23].

Reported postoperative complications ranging from discomfort and lip tightness to swelling, bruising, paresthesia, and rarely mucocele formation are generally mild and self‐limiting. Overall, LR remains a safe, conservative, and effective adjunct or alternative to orthognathic surgery in carefully selected patients; nevertheless, larger prospective studies with extended follow‐up are required to better define long‐term stability and predictors of relapse.

This case underscores the importance of addressing both hard‐ and soft‐tissue etiologies in a staged, evidence‐based manner. The use of digital planning, guided surgery, and modern mucogingival techniques allowed for optimal control over both esthetic and functional parameters. From a biomechanical perspective, preserving the attached gingiva and minimizing tension on closure likely contributed to the favorable healing and minimal scarring observed.

### 3.1. Limitations

Although the 5‐year follow‐up strengthens the longitudinal value of this current case report, conclusions regarding long‐term stability of LR with selective myectomy should be interpreted cautiously. Gingival display measurements were based on standardized photographic analysis, which may be subject to minor variability in head position and smile reproducibility. Skeletal assessment relied on clinical and conventional radiographic evaluation without 3D imaging. Additionally, muscle healing and reattachment were not objectively quantified. Future controlled studies with larger samples and standardized outcome measures are necessary to validate these findings.

## 4. Conclusion

A comprehensive diagnostic approach is crucial in managing EGD involving skeletal, dental, and muscular factors. In this case, digitally guided ECL and LR achieved substantial esthetic improvement over 5 years, with minor recurrence (2–3 mm) but sustained patient satisfaction. The digital workflow and 3D‐printed guide enhanced surgical precision, while staged management ensured biological stability. Despite minor relapse, long‐term results remained satisfactory, highlighting the efficacy of this minimally invasive, technology‐integrated approach. Incorporating digital planning into periodontal and mucogingival surgeries offers a predictable, patient‐centered pathway for esthetic rehabilitation, though further studies are needed to standardize protocols and optimize long‐term outcomes.

## Author Contributions

Hytham N. Fageeh contributed to the conception and design of the study, clinical management and surgical procedures, data acquisition, analysis and interpretation, and drafting and revision of the manuscript.

## Funding

The author received no financial support for the research, authorship, and/or publication of this article.

## Disclosure

The author approved the final version of the manuscript.

## Ethics Statement

Ethical approval was obtained by the Ethics Committee of the Scientific Research Unit at the College of Dentistry (Approval Number CODJU‐2220F). The study was conducted in accordance with the Declaration of Helsinki and institutional ethical standards.

## Consent

Written informed consent was obtained from the patient for treatment and publication of clinical information and accompanying images.

## Conflicts of Interest

The author declares no conflicts of interest.

## Data Availability

All relevant data supporting the findings of this case report are included within the article. Additional information is available from the corresponding author upon reasonable request, while maintaining patient confidentiality.
